# Octenyl Succinic Anhydride-Modified Starch Attenuates Body Weight Gain and Changes Intestinal Environment of High-Fat Diet-Fed Mice

**DOI:** 10.3390/foods11192980

**Published:** 2022-09-23

**Authors:** Jia-Ping Chen, Kuerbanjiang Maierheba, Ying Zhang, Hui Cheng, Binbin Lin, Pan Yue, Le-Hua Wang, Feng-Zhi Liu, Jun-Wen Shi, Zhong-Xiao Wan, Xiao-Ping Wang, Jianteng Xu, Li-Qiang Qin, Yanjie Bai

**Affiliations:** 1 Department of Nutrition and Food Hygiene, School of Public Health, Soochow University, No. 199 Ren’ai Road, Dushu Lake Higher Education District, Suzhou 215123, China; 2 Suzhou Industrial Park Centers for Disease Control and Prevention, No. 200 Su’hong Xi Road, Suzhou 215123, China; 3 Grain Processing Corporation, 1600 Oregon Street, Muscatine, IA 52761, USA; 4 Department of Chemical Engineering, Hebei University of Technology, No. 5340 Xi’ping Road, Beichen District, Tianjin 300000, China

**Keywords:** octenylsuccinate starch, high-fat diet, fecal short-chain fatty acids, gut microbiota, gas chromatography, 16S rDNA sequencing

## Abstract

Effects of octenylsuccinate (OS) starch on body composition and intestinal environment in high-fat diet-fed mice were investigated. C57BL/6J mice were treated with a regular-fat (RF) diet, a high-fat (HF) diet, or a high-fat diet supplemented with OS starch (HFOSS). Fecal short-chain fatty acids (SCFAs) were quantified using gas chromatography, and the fecal microbiota profile was analyzed by 16S rDNA sequencing. One-way ANOVA and metastats analysis were performed for statistical analysis. After 22 weeks of feeding, mice in the HFOSS group had significantly lower body weight, body fat, liver weight, and cumulative food intake than those in the HF group but higher than that of the RF group. Fecal total SCFA, acetic, propionic, and butyric acid concentrations were significantly higher in the HFOSS group than that in the HF and RF groups. OS starch intervention increased the relative abundance of *Parabacteroides*, *Alistipes*, and *Ruminiclostridium_5* and decreased that of *Tyzzerella*, *Oscillibacter*, *Desulfovibrio*, and *Anaerotruncus* compared with the RF and HF groups. The relative abundance of *Lachnospiraceae_UCG-006* in the HFOSS group was lower than that in the HF group but higher than that in the RF group. In conclusion, OS starch prevents fat accumulation in high-fat diet-fed mice and might provide potential health benefits due to its fermentability in the gut and its ability to regulate gut microbial community structure.

## 1. Introduction

Octenylsuccinate (OS) starch generally is prepared by esterification of octenylsuccinicanhydride (OSA) and starch under mild alkaline conditions [1,2]. OS starch was originally developed as a food emulsifier by Caldwell and Wurzburg [3]. Compared to protein emulsifiers, OS starch is less sensitive to pH, ion concentration, and temperature in food emulsions [4]; therefore, it is widely used in beverages, creams, and salad dressings [2,5,6]. OS starch is also commonly used as a microencapsulated wall material, thickener, and fat replacer [5,6].

In recent years, beneficial nutritional properties were reported for OS starch. Wolf et al. [7] found that OS starch increased blood glucose levels much more slowly than the same amount of glucose in healthy adults. Similarly, studies reported that OS starch could reduce glycemic and insulinemic responses in human subjects compared to glucose [8,9]. A recent study showed that OSA-modified starch effectively promoted satiety in mice, which was accompanied by increased plasma levels of glucagon-like peptide 1 (GLP-1) and peptide YY (PYY) [10].

A diet rich in saturated fatty acids is one of the major causes of obesity and related metabolic disorders [11,12,13]. Resistant starch (RS) has been suggested to prevent the deleterious effects of a high-fat (HF) diet. RS supplementation was shown to attenuate body weight gain [14,15] and regulate glucose and lipid metabolism [16,17,18]. The beneficial effects of RS are partially attributed to the modulation of gut microbiota flora disorders [15,19,20,21]. RS was suggested to selectively stimulate the growth of probiotics and inhibit the growth of pathogenic taxa [22,23,24,25,26].

It has been suggested that the digestibility of starch is reduced after OSA modification. Studies showed that OSA-modified potato, corn, cassava, waxy maize, and banana starches significantly increased the content of slowly digestible starch (SDS) and RS [27,28,29,30,31,32,33,34,35,36,37,38,39,40,41]. Moreover, a hydrogen respiration experiment suggested that OS starch could partially escape digestion and absorption in the small intestine and be fermented by bacteria in the large intestine [9]. An in vitro study showed that OS starch was fermented into significant amounts of short-chain fatty acids (SCFAs), particularly butyric acid [42]. We postulated that the fecal SCFA composition and gut microbiota structure might be altered by the resistant starch fraction of OS starch. However, the effects of OS starch on the gut microenvironment have not been investigated.

In this study, mice were fed three different diets. For the regular-fat (RF) diet, 10% of energy was provided from fat. The HF diet had components similar to those of the RF diet but with higher energy density. The HF diet supplemented with OS starch (HFOSS) was designed to have a similar energy density as the HF diet. The energy provided by fat, protein, and carbohydrate was adjusted to be the same for the HF and HFOSS diets. Dietary fiber was derived from OS starch in the HFOSS diet and accounted for 10% (wt.%) of the total weight of the diet. In comparison, cellulose was the dietary fiber source for the RF and HF diets and accounted for 5% (wt.%) of the diet weight. The body composition of the mice was measured during the experiment. The composition of SCFAs in mouse feces was determined by gas chromatography (GC). The fecal microbiota structure was analyzed using 16S rDNA high-throughput sequencing. This study evaluated the health benefits of OS starch and revealed the impact of OS starch on fecal microbiota structure and SCFA composition in mice under the stress of a high-fat diet.

## 2. Materials and Methods

### 2.1. Materials

Sweet potato starch was isolated by settling pureed sweet potato (Jishu-25, Qinhuangdao, China) in water after fiber removal. The 2-Octen-1-ylsuccinic anhydride, porcine pancreatin, and D-glucose were purchased from Sigma–Aldrich Chemical Co. (Shanghai, China). GC standards of acetic, propionic, butyric, isobutyric, valeric, and isovaleric acid were purchased from Macklin Biochemical Co., Ltd. (Shanghai, China). The 2-ethylbutyric acid was obtained from Aladdin Biochemical Technology Co., Ltd. (Shanghai, China). Insulin (3 mL, 300 IU) was purchased from Lilly, France (Suzhou, China). The neutral formalin solution (10%, *v*/*v*) was from Wuhan Servicebio Technology Co., Ltd. (Wuhan, China). All chemicals were of analytical grade, except for those mentioned specifically. Eight-week-old male C57BL/6J mice, an obesity-prone breed, were provided by Soochow University Laboratory Animal Center (Suzhou, China). A normal chow diet was provided by the Double Lion Experimental Animal Feed Technology Co., Ltd. (Suzhou, China). Experimental diets were obtained from Medicience Ltd. (Yangzhou, China).

### 2.2. Methods

#### 2.2.1. Preparation and Characterization of OS Starch

OS starch was prepared according to the method described by Bai and Shi [43]. Englyst method was used to estimate the digestibility of OS starch in vitro and performed as described in the literature [44,45]. The content of rapidly digestible starch (RDS), SDS, and RS was calculated using the Equations (1)–(3), respectively:

(1) $$\mathrm{RDS}=\frac{(G20\times 0.9)}{W}$$

(2) $$\mathrm{SDS}=\frac{(G120-G20)\times 0.9}{W}$$

(3) RS=100%−(RDS+SDS)

where G20 and G120 are the amount of glucose (g) measured after enzyme hydrolysis for 20 and 120 min, respectively; W is the dry weight of OS starch (g).

The degree of substitution (DS), the weight percentage of the OS group in OS starches (%OS), and reaction efficiency (RE) were determined using NMR spectrometry, as previously reported [46]. DS, %OS, and RE were calculated using Equations (4)–(6), respectively.

(4) $$\mathrm{DS}=\frac{I_{0.93}/3}{I_{5.36}+I_{5.10}+I_{4.93}+I_{4.59}-I_{5.52}}$$

where I_0.93_ is the integral of the methyl proton; I_5.__52_ is the integral of one of the alkene protons; I_5.__36_ is the integral of the internal H-1 of α-1,4-linked glucose units plus one of the alkene protons; I_5.10_ is the integral of the H-1 of the α-anomeric reducing end; I_4.93_ is the integral of the H-1 of the branching point; and I_4.59_ the integral of the H-1 of the β-anomeric reducing end.

(5) $$\%\mathrm{OS}=\frac{210\times \mathrm{DS}}{209\times \mathrm{DS}+162}$$

(6) $$\mathrm{RE}=\frac{\%\mathrm{OS}\;\mathrm{of}\;\mathrm{OS}\;\mathrm{starch}}{\%\mathrm{OSA}\;\mathrm{added}\;\mathrm{to}\;\mathrm{the}\;\mathrm{starch}}$$

#### 2.2.2. Animal Experiment

Mice were raised in a lab with a 12 h light/dark cycle at 24 °C and humidity of 60%. All procedures followed the National Institutes of Health’s Guide for the Care and Use of Laboratory Animals. The experiment was approved by the Soochow University Animal Ethics Committee with an approval number of 201705A127.

All mice were provided ad libitum access to a normal chow diet and water for 12 days for acclimatization. The mice were then randomly divided into three groups (10 mice per group, 5 mice per cage) and fed an RF diet (D12450J), HF diet, and HFOSS diet, respectively. From weeks 1 to 9, the HF and HFOSS diets were based on a high-fat diet of D12451 (Research Diets, Inc., New Brunswick, NJ, USA). Details of the diet are described in Supplemental Materials Table S1. From weeks 10 to 22, HF and HFOSS were based on a high-fat diet of D12492 (Research Diets, Inc., New Brunswick, NJ, USA). Formulations of the diets are described in Table 1. All diets were sterilized using Co60 radiation.

#### 2.2.3. Body Composition, Lee Index, and Food Intake of Mice Fed Different Diets

Every mouse was weighed weekly. The food and energy intake of mice were recorded weekly. After 22 weeks, all mice were sacrificed. The body weight, length of the mice, and the weight of liver and epididymal and perirenal adipose tissues were recorded. The Lee index, fat ratio, and liver ratio were calculated using Equations (7)–(9), respectively:

(7) $$\mathrm{Lee index}=\sqrt[{3}]{\frac{W}{L}}$$

W is the body weight of the mice (g), and L is the length of the mouse body from the nose to the anus (cm).

(8) $$\mathrm{Fat}\;\mathrm{ratio}=\frac{W_{e}+W_{p}}{W}$$

W_e_ is the epididymal adipose weight (g), W_p_ is the perirenal adipose weight (g) of mice, and W is the body weight of mice (g).

(9) $$\mathrm{Liver}\;\mathrm{ratio}=\frac{W_{l}}{W}\times 100\%$$

W_l_ is the liver weight (g), and W is the body weight of mice (g).

#### 2.2.4. Blood Samples Preparation

After the mice were sacrificed, blood samples were collected with an anticoagulant of heparin. Immediately after collection, blood samples were centrifuged at 3000× *g* for 20 min at 4 °C (5427R; Eppendorf, Hamburg, Germany). Plasma was removed and stored at −80 °C before analysis. GLP-1was measured using a GLP-1 ELISA kit (Millipore Corp., Billerica, MA, USA).

#### 2.2.5. Intraperitoneal Glucose Tolerance Tests (IPGTT)

At week 22, before the mice were sacrificed, the IPGTT was performed. Glucose (40%, *v*/*v*) at 2 g/kg body weight was intraperitoneally injected into overnight-fasted mice. Before injection and 30, 60, and 120 min after injection, the blood glucose levels of mice were measured from the tail using a glucometer (Roche Diagnostics, Mannheim, Germany). The area under the curve (AUC) for glucose was estimated using the trapezoid method [47].

#### 2.2.6. Insulin Tolerance Test (ITT)

At week 22, on the second day of the IPGTT test, the ITT was performed before the mice were sacrificed. Insulin of 0.75 U/kg based on the body weight of mice was injected. The blood glucose levels of mice before and 30, 60, and 120 min after injection were measured from the tail using a glucometer as described above.

#### 2.2.7. Histopathological Examination

Mouse tissues were stained with hematoxylin–eosin (H&E) and Oil Red O for histopathological examination. The samples were prepared by Wuhan Servicebio Technology Co., Ltd. (Suzhou, Jiangsu, China). Mouse colons and livers were placed in a 10% neutral formalin solution and fixed in paraffin. A pathology slicer (RM2016; Leica Instrument Co., Ltd., Shanghai, China) was used to slice the fixed tissues into 4 μm-thick sections. The cells were then stained with an H&E solution. Frozen liver tissues were sectioned at a thickness of 8–10 µm using a cryostat (CryoStar NX50; Thermo Scientific, Waltham, MA, USA) and stained with an Oil Red O solution. Slides were observed under a light microscope (Eclipse E100; Nikon, Tokyo, Japan). The detailed method is described in Section S2.

#### 2.2.8. Collection of Mice Feces

At week 20, the mice were individually placed in metabolism cages. Fresh samples were collected at night (from 6 pm to 9 pm) over a 3-day period. The fecal weight of one mouse collected in 3 h was recorded. After collection, the samples were stored at −80 °C for further analysis.

#### 2.2.9. Determination of Fecal Fat Content

The excreted fecal fat content was analyzed as reported with some modifications [48]. The frozen feces were freeze-dried. The feces weight before and after freeze-dry was recorded, and the fecal moisture content was calculated. Freeze-dried feces (approximately 0.1 g) were ground by a mortar and pestle. A mixture of chloroform and methanol (1 mL, 2:1, *v*/*v*) was added to feces and magnetically stirred at room temperature for 24 h. Aliquots were centrifuged at 5000 rpm for 20 min (5427R; Eppendorf, Hamburg, Germany). The chloroform phase was collected and dried under nitrogen gas. The dried fat residue (W_fat_) was weighed. The fecal fat content (C_fat_), the 3 h excreted fecal fat weight (W_fat-3h_), and the fecal moisture content (MC) were calculated using Equations (10)–(12), respectively.

(10) $$\mathrm{Fecal}\;\mathrm{fat}\;\mathrm{content}\;(C_{\mathrm{fat}},\;\%)=\frac{W_{fat}}{W}\times 100\%$$

*W_fat_* is the weight of fat residue (g), and *W* is the fecal weight, as is (g).

(11) $$\mathrm{Excreted}\;\mathrm{fecal}\;\mathrm{fat}\;\mathrm{weight}\;(W_{\mathrm{fat}-3h},\;g)=C_{fat}\times W_{3h}$$

*W*_3_*_h_* is the weight of feces, as is from one mouse collected in 3 h (g).

(12) $$\mathrm{Fecal}\;\mathrm{moisture}\;\mathrm{content}(\mathrm{MC},\;\%)=\;\frac{W-W_{dry}}{W}\times 100\%$$

*W* is the fecal weight, as is (g), and *W_dry_* is the fecal weight after freeze drying (g).

#### 2.2.10. Determination of the Short-Chain Fatty Acid (SCFA) Concentrations in Mice Feces

SCFAs were determined, as previously reported, with some modifications [49]. Mice feces collected at week 20 were thawed at room temperature for 30 min before preparation. Feces (0.1 g) was mixed with 15 μL of 2-ethylbutyric acid (2 µL/mL) using a tissuelyser (JXFSTPRP-48; Jingxin Industrial Development Co., Ltd., Shanghai, China) at 70 Hz/min for 3 min. Phosphoric acid (1 mL; 0.5%, *v*/*v*) was then added to the suspension and dispersed with the tissuelyser at 70 Hz/min for 5 min. The suspension was vortexed for 1 min and centrifuged at 17,949× *g* for 10 min at 25 °C. The supernatant was collected, mixed with ethyl acetate (1 mL), vortexed for 2 min, and centrifuged at 17,949× *g* for 10 min. The upper layer was collected and filtered using a 0.22 mm filter for analysis.

The GC system (7890A; Agilent Technologies, Palo Alto, CA, USA) was equipped with a flame ionization detector (FID) and a fused silica capillary column (DB-WAX, 30 m 0.25 mm i.d, 0.25 μm; Agilent, Palo Alto, CA, USA). Helium was used as the carrier gas at a flow rate of 1 mL/min. The oven temperature profile was set at 90 °C for 1 min, raised to 150 °C at a heating rate of 15 °C/min, held at 150 °C for 1 min, increased to 170 °C at a heating rate of 5 °C/min, and then ramped to and maintained at 240 °C for 5 min. The temperatures of the injection port and FID were set to 220 °C and 300 °C, respectively. The injection volume used was 1 µL. Acetic, propionic, butyric, isobutyric, valeric, and isovaleric acids were quantified from the peak area using an internal standard (2-ethylbutyric acid) calibration curve. Data were handled using OpenLab CDS (Agilent Technologies, Palo Alto, CA, USA).

#### 2.2.11. Fecal Sample Preparation and 16S rDNA Gene Sequencing

Fecal samples (0.1 g) were ground with liquid nitrogen by a mortar and pestle to a fine powder. DNA extraction was conducted in accordance with the instructions of the DNeasyPowerSoil Kit (Cat No. 47014; Qiagen, Hilden, Germany). A spectrophotometer (Nanodrop 2000, Thermo Scientific, Waltham, MA, USA) was used to measure the concentration of DNA. The 16S rDNA sequencing was performed by GENEWIZ, Inc. (Suzhou, Jiangsu, China) using the Illumina MiSeq system (Illumina, Inc., San Diego, CA, USA). Amplicon generation, library preparation, and data analysis followed the methods described in previous studies [50,51].

The 16S rDNA data analysis was performed by the QIIME data analysis package using effective sequences. The sequences were grouped into operational taxonomic units (OTUs). Alpha diversity indices of ACE, Chao 1, Shannon, and Simpson were calculated from rarefied samples. Beta diversity was calculated using the principle components analysis (PCA). The variation between groups was calculated using Anosim.

#### 2.2.12. Statistical Analysis

GraphPad Prism 7 (GraphPad Software Inc., La Jolla, CA, USA) was used for statistical analyses. One-way ANOVA was used to assess differences in body composition, food and energy intake, glucose and insulin tolerance, fecal fat content, and SCFAs composition. Tukey’s multiple comparison test was performed to determine significant differences at the 95% confidential level. The metastats analysis was performed to determine significant differences in the gut microbiota between the groups. Data are presented as the mean ± standard deviation (SD), and a *p*-value < 0.05 was considered to be statistically significant.

## 3. Results and Discussion

### 3.1. Effects of OS Starch on Body Composition and Food and Energy Intake of Mice Fed an HF Diet

The RDS, SDS, and RS contents of the OS starch were 28.9%, 35.3%, and 37.1%, respectively. The DS of the OS starch was 0.0173; %OS and RE of the OS starch were 2.19% and 73%, respectively. The OS starch has a significant amount of resistant starch.

**Figure 1:**
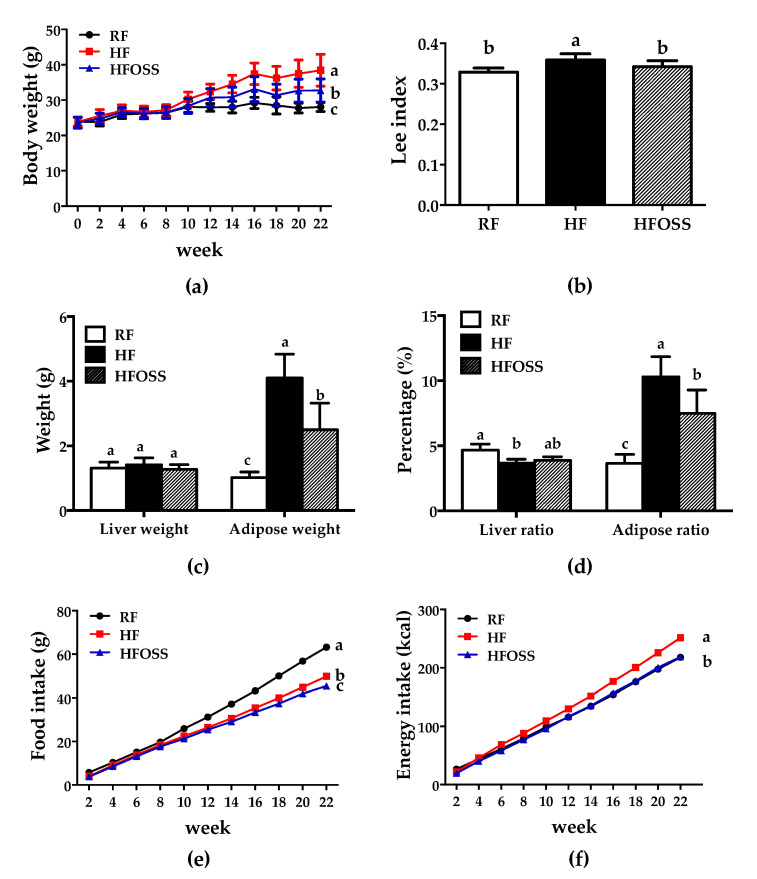
(**a**) Body weight, (**b**) Lee index, (**c**) liver and adipose weight, (**d**) liver and adipose ratio, (**e**) cumulative food intake, and (**f**) cumulative energy intake of mice fed a regular-fat diet (RF), high-fat diet (HF) and high-fat diet supplemented with OS starch (HFOSS). Values with different letters indicate significant differences between groups (*p* < 0.05).

During the first 8 weeks, the body weights of the mice in RF, HF, and HFOSS groups were similar. The HF diet did not induce obesity in mice. Therefore, mice in the HF and HFOSS groups were fed diets with different formulations (Table 1). Thereafter, mice in the HF and HFOSS groups grew rapidly. At the end of the experimental period, the body weight of mice in the HF group was significantly higher than that of mice in the RF and HFOSS groups (Figure 1a). Similar results were observed for the Lee index, adipose weight, and adipose ratio (Figure 1b–d).

OS starch supplementation reduced fat accumulation in mice fed an HF diet. Mice in the HFOSS group had significantly lower body weight, Lee index, adipose weight, and adipose ratio than those in the HF group (Figure 1a–d). The cumulative food intake and energy intake of mice in the HFOSS group were significantly lower than those in the HF group (Figure 1e,f). Mice in RF and HFOSS groups had similar cumulative energy intake (Figure 1f).

It has been suggested that RS aids weight management because of its low energy density, strong satiety effect, and enhancement of energy expenditure or fat oxidation [52]. A previous study reported that supplementing OS starch to mice under a regular fat diet did not significantly change their body weight [27]. For the first time, we observed that OS starch effectively prevented fat accumulation in mice fed an HF diet. Our results imply that OS starch might suppress the appetite of mice, reduce their energy intake, and thus prevent their body weight growth. A recent study reported that OSA-modified high amylose corn starch reduced food intake in mice in the short term and increased satiety-related intestinal hormones PYY and GLP-1 [10]. However, in this study, we did not observe a significant increase in plasma GLP-1 levels (Figure S1). GLP-1 and PYY are satiety hormones secreted by intestinal enteroendocrine L-cells [53]. Some resistant starches were found to stimulate the secretion of GLP-1 or PYY [54,55,56,57,58], but the results are not always consistent [53]. It was proposed that the secretion of GLP-1 may be affected by the structure of resistant starch [10]. The effect of OS starch on appetite and its related mechanisms is an interesting subject and can be investigated in the future.

### 3.2. Effects of OS Starch on Glucose and Insulin Tolerance in Mice Fed a High-Fat Diet

**Figure 2:**
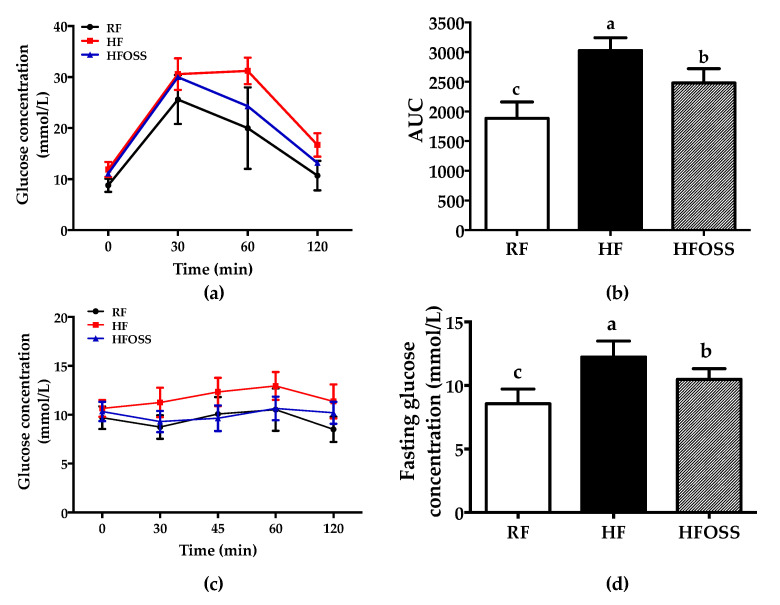
Glucose and insulin tolerance of mice fed a regular-fat (RF) diet, high-fat (HF) diet, and high-fat diet supplemented with OS starch (HFOSS). (**a**) Blood glucose response of mice under intraperitoneal glucose tolerance test (IPGTT); (**b**) area under the curve (AUC) of the IPGTT; (**c**) blood glucose response of mice under insulin tolerance test (ITT); (**d**) fasting blood glucose concentration of mice. Values with different letters indicate significant differences between groups (*p* < 0.05).

The IPGTT results suggested that blood glucose peaked at 30 min for mice in the RF and HFOSS groups but peaked at 60 min for mice in the HF group (Figure 2a). The AUC of mice in the HFOSS group was significantly lower than that of the HF group but higher than that of the RF group (Figure 2b).

Insulin tolerance appeared to be impaired for mice in the HF group, as their blood glucose levels increased after insulin injection. In comparison, mice in the HFOSS and RF groups showed better insulin sensitivity (Figure 2c). It was also observed that mice fed the HFOSS diet had lower fasting blood glucose levels than those fed the HF diet (Figure 2d).

Our results suggest that OS starch may improve glucose tolerance and insulin sensitivity in mice fed an HF diet. Similar results were reported in the literature. Compared to the same amount of glucose, healthy adults were found to have attenuated postprandial glycemic excursion after consuming OS starch [7]. It has been proposed that OSA modification increases the content of slowly digestible starch (SDS) [8]. SDS releases glucose slowly in the small intestine, which may be helpful in improving glucose tolerance [59]. In addition, improved glucose metabolism might be related to the fermentation of OS starch in the gut. OS starch can provide substrates to gut microbial and generate SCFAs. SCFAs can help improve insulin sensitivity via several pathways [60].

### 3.3. Effects of OS Starch on the Histology of Liver and Colon Tissues

**Figure 3:**
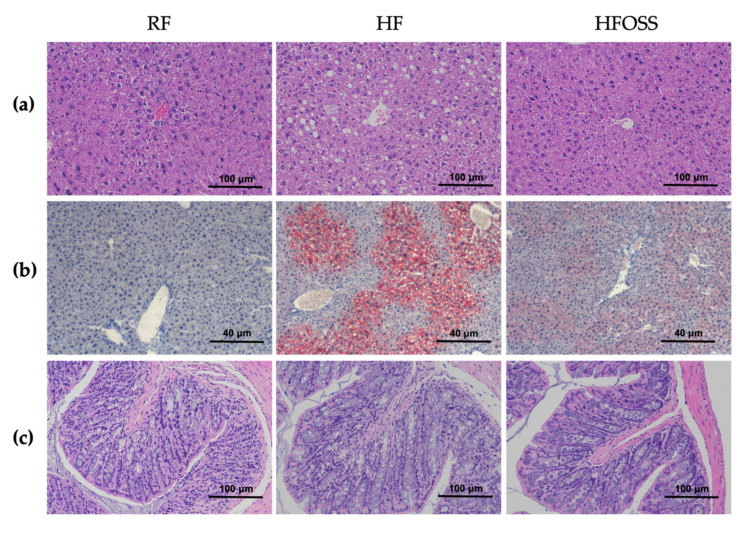
Histopathological analysis of mice fed a regular-fat diet (RF), high-fat diet (HF), and high-fat diet supplemented with OS starch (HFOSS). (**a**) Hematoxylin–eosin (H&E) staining of liver tissues; (**b**) Oil Red O staining of liver tissues; (**c**) H&E staining of colon tissues.

Compared with the RF group, more lipid droplets were observed in the livers of HF diet-fed mice, accompanied by cytoplasmic ballooning and inflammation (Figure 3a). The symptoms were improved in the HFOSS group. The liver tissues of OS starch-treated mice showed fewer lipid droplets and fewer inflammatory cells, as confirmed by Oil Red O staining (Figure 3b). Liver histology suggested that OS starch could prevent steatosis and improve inflammation in the liver.

Resistant starch was shown to improve liver steatosis in mice fed an HF diet [15,17,18]. It was suggested that RS increases SCFAs in the gut, which modulates liver protein levels involved in lipid metabolism and ameliorates hepatic steatosis [18]. SCFAs were suggested to improve fat metabolism and accumulation by activating the AMP-activated protein kinase (AMPK) and peroxisome proliferator-activated receptor (PPAR) signaling pathways [61].

The colons of mice in the three groups showed an intact colon structure (Figure 3c). This result indicated that the HF and HFOSS did not change colon histology in this study. Resistant starch was reported to have an anti-inflammation effect in the colon of mice fed an HF diet [62]. It was reported that some food emulsifiers might promote diseases related to gut inflammation [63]. In our study, we did not observe any negative impact of OS starch on colon histopathology. OS starch may be different from detergent-like emulsifiers in the gut environment, which can be further investigated.

### 3.4. Effects of OS Starch on the Fecal Fat Content of Mice Fed a High-Fat Diet

**Figure 4:**
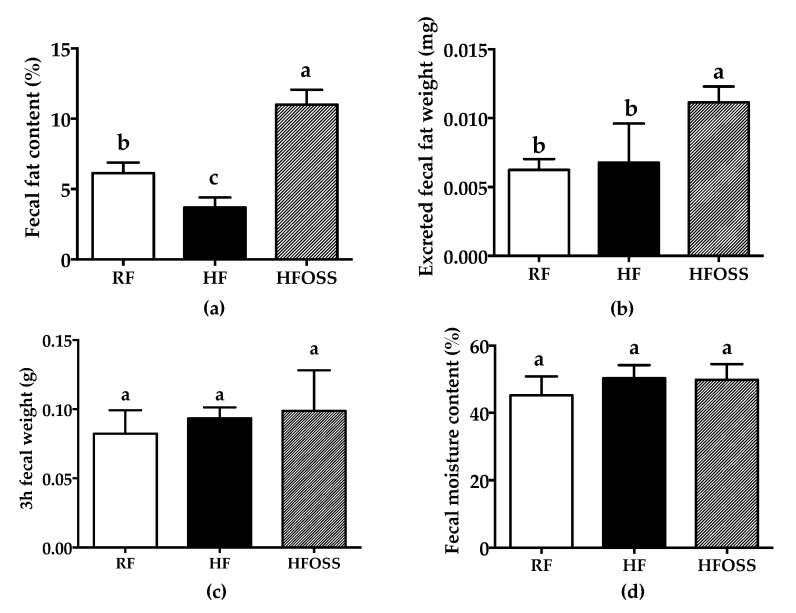
(**a**) Fecal fat content (weight%, based on the fecal weight as is); (**b**) excreted fecal fat weight per mouse during a 3 h collection period; (**c**) fecal weight of one mouse during a 3 h collection period; (**d**) fecal moisture content (%). Mice were fed a regular-fat diet (RF), high-fat diet (HF), and high-fat diet supplemented with OS starch (HFOSS). Values with different letters indicate significant differences between groups (*p* < 0.05).

The fecal fat content of mice in the RF, HF, and HFOSS groups was 6.1%, 3.7%, and 11.0%, respectively (Figure 4a). Compared with that of the RF group, the fecal fat content of the HF group was significantly decreased. OS starch supplementation significantly increased the fecal fat content of HF diet-fed mice (*p* < 0.05). The fecal weight excreted at 3 h and the fecal water content was similar for all three groups (Figure 4c,d). Therefore, the fat excreted within 3 h was significantly higher in the HFOSS group than in the RF and HF groups (Figure 4b).

Previous studies showed that RS can increase fecal fat content in mice [24,48], which is consistent with our results. OS starch is amphiphilic and is known to encapsulate oily substances, such as dietary fat. It is possible that OSA-starch exerts an encapsulating effect on fat particles and hinders their digestion and absorption in the gut. Our results suggest that the weight control benefit of OS starch may be a result of reduced fat absorption.

### 3.5. Effects of OS Starch on the Fecal SCFA Composition of Mice Fed a High-Fat Diet

**Figure 5:**
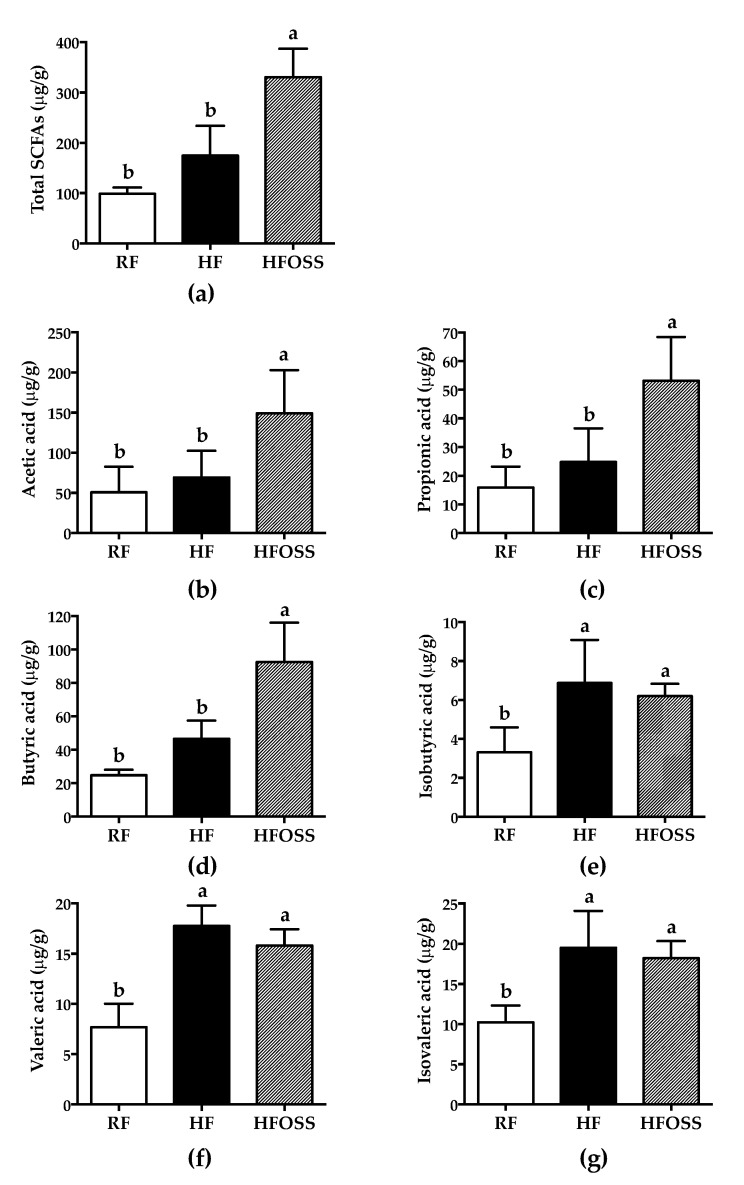
Fecal short-chain fatty acids (SCFAs) concentrations of mice fed a regular-fat diet (RF), high-fat diet (HF), and high-fat diet supplemented with OS starch (HFOSS). (**a**) Total SCFAs, (**b**) acetic acid, (**c**) propionate acid, (**d**) butyric acid, (**e**) isobutyric acid, (**f**) valeric acid, and (**g**) isovaleric acid. Values with different letters indicate significant differences between groups (*p* < 0.05).

Six short-chain fatty acids were detected in mouse feces, with acetic acid being the dominant SCFA (Figure 5). Mice fed the HF and RF diets had similar total SCFAs concentrations. These results suggest that these two diets had similar gut fermentability. In comparison, OS starch significantly increased the total fecal SCFAs, acetic acid, propionic acid, and butyric acid concentrations compared to the RF and HF diets (Figure 5a–d), which was consistent with the in vitro fermentation results [42]. This is the first report of the fermentation characteristics of OS starch in vivo. These results suggested that OS starch had relatively good gut fermentability and could change the fecal SCFA pattern of mice fed an HF diet.

Resistant starch as a complex carbohydrate is the preferred substrate for large bowel microflora fermentation, and SCFAs are the major metabolic products [64]. Different types of RS were reported to increase the content of SCFAs in the cecum of rats, accompanied by decreased weight gain [17,65,66,67,68]. Although the effect of OS starch on fecal SCFAs concentration is less investigated, together with the in vitro study [42], OS starch appears to be a fermentable carbohydrate in the colon and produces primally acetic, propionic, and butyric acids. OS starch may be a preferred substrate for some bacteria, which is discussed in detail in the next section. We proposed that the positive effect of OS starch on body weight management may be partially attributed to its SCFA-generating effect. Human and animal studies demonstrated that SCFA supplementation could reduce body weight gain and improve glucose homeostasis and insulin sensitivity [69,70,71,72]. The increased gut acetic and propionic acids concentration may improve insulin sensitivity and blood glucose [73]. In addition, butyric acid was suggested to provide energy for intestinal epithelial cells, maintain homeostasis of the intestinal environment, inhibit the inflammatory response, and reduce appetite [70].

### 3.6. Effects of OS Starch on Fecal Microbiota Structure

#### 3.6.1. Gut Microbiota Composition at the Phylum Level

**Figure 6:**
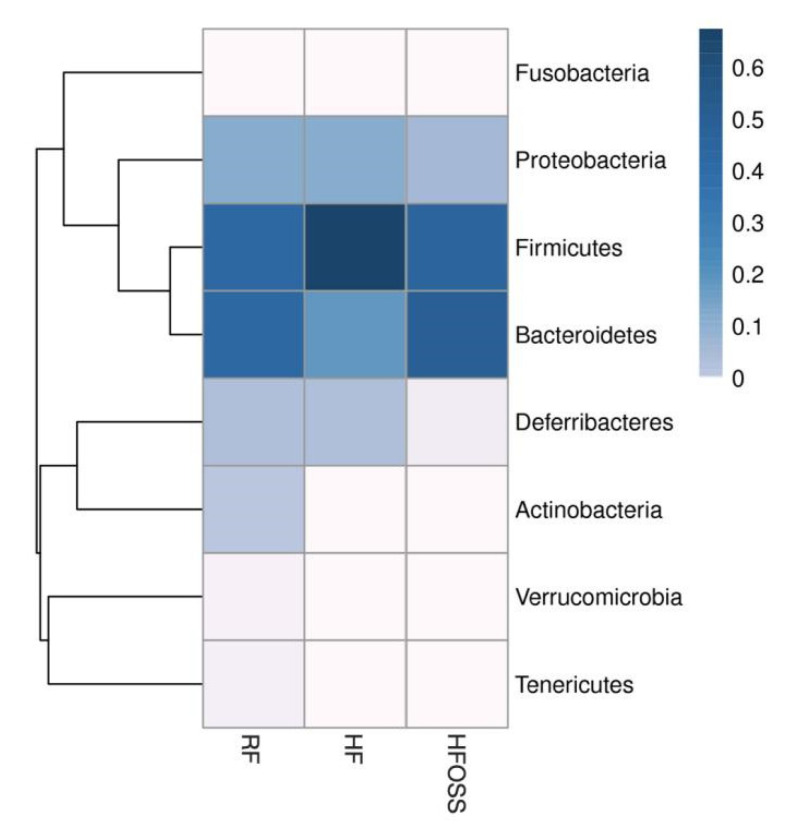
Phylum distribution heatmap of the fecal microbiota of mice fed a regular-fat diet (RF), high-fat diet (HF), and high-fat diet supplemented with OS starch (HFOSS). The colors in the heatmap represent the relative abundance of the corresponding phylum in the corresponding group.

*Firmicutes*, *Bacteroidetes*, and *Proteobacteria* were the dominant bacterial phyla in the three groups, which is consistent with the literature [74]. However, the relative abundance of the three bacterial phyla varied (Figure 6). Mice fed an HF diet had a significantly higher relative abundance of *Firmicutes*. The ratios of *Firmicutes* to *Bacteroides* (F/B) in the RF, HF, and HFOSS groups were 1.29, 11.15, and 1.26, respectively. The HF diet significantly increased the F/B ratio compared to the RF diet, and this trend was reversed by OS starch intervention. The HFOSS group also had a lower relative abundance of *Proteobacteria* and *Deferribacteres* than the RF and HF groups.

Several studies reported that resistant starch supplementation decreases the F/B ratio [75,76,77]. The F/B ratio is generally positively correlated with body weight and inflammatory conditions [78,79,80,81,82]. It was proposed that *Firmicutes* are more effective in promoting calorie absorption than *Bacteroidetes*, and subjects who had an increased abundance of *Firmicutes* had increased calorie absorption, resulting in weight gain [83]. *Proteobacteria* is another dominant bacterial phylum in mouse feces. Both in vivo and in vitro studies showed that RS could decrease the abundance of *Proteobacteria* [77,84], which is consistent with our results. *Proteobacteria* may reflect microecological disorders or instability of the intestinal microbial community structure [85,86,87]. Therefore, OS starch may promote microbiota stability in mice fed an HF diet.

#### 3.6.2. Gut Microbiota Composition at the Genus Level

**Figure 7:**
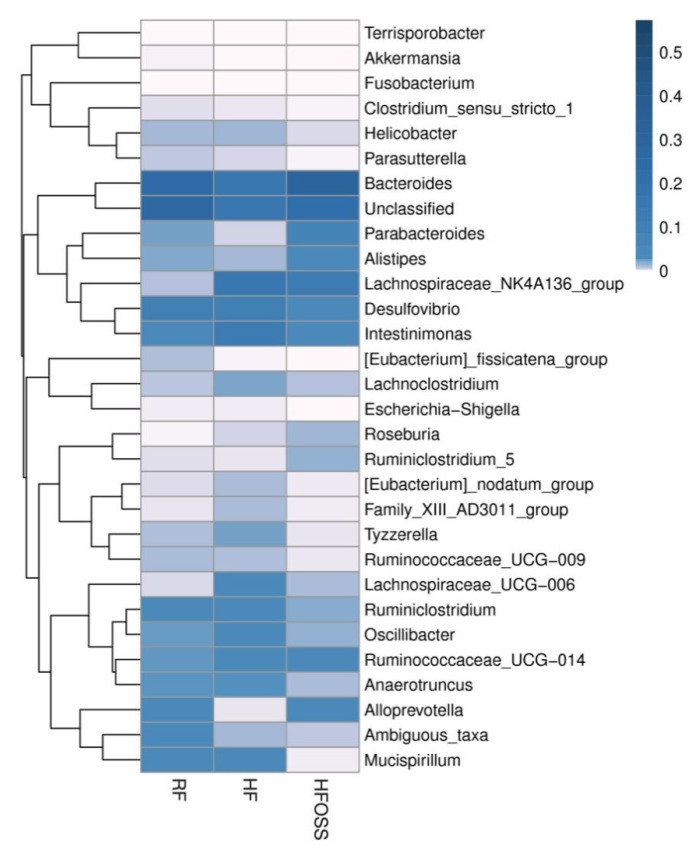
Genus distribution heatmap of the fecal microbiota of mice fed a regular-fat diet (RF), high-fat diet (HF), and high-fat diet supplemented with OS starch (HFOSS). The colors in the heatmap represent the relative abundance of the corresponding genus in the corresponding group.

The fecal microbiota structure of mice fed different diets differed at the genus level (Figure 7). The three dominant bacterial genera in the RF group were *Bacteroides*, *Desulfovibrio,* and *Intestinimonas*. The three dominant bacteria in the HF group were *Bacteroides*, *Lachnospiraceae_NK4A136_group*, and *Intestinimonas*. *Bacteroides*, *Lachnospiraceae_NK4A136_group*, and *Parabacteroides* were the three most dominant genera in the HFOSS group. Metastatic analysis suggested that the HF diet significantly increased the relative abundance of *Lachnospiraceae_NK4A136_group* and *Lachnospiraceae_UCG-006* compared to the RF diet (*p* < 0.05). Our study results are consistent with previously reported results [88,89]. Compared with the HF diet, the OS starch intervention significantly increased the relative abundance of *Parabacteroides*, *Alistipes*, and *Ruminiclostridium_5* and decreased the relative abundance of *Desulfovibrio*, *Oscillibacter*, *Anaerotruncus*, *Lachnospiraceae_UCG-006* and *Tyzzerella* (Figure S2).

The *Lachnospiraceae* family belongs to *Clostridial cluster XIVa* of the phylum *Firmicutes* [90,91]. The increased relative abundance of *Firmicutes* in the HF group was partially attributable to the increased abundance of the *Lachnospiraceae* family. The physiological effects of the *Lachnospiraceae_ NK4A136*_group and *Lachnospiraceae UCG-006* remain unclear. *Lachnospiraceae UCG-006* has been suggested to be an SCFA-producing genus, especially acetic acid [92].

OS starch had a positive effect on the fecal microbiota structure of HF diet-fed mice. *Parabacteroides* and *Alistipes* were suggested to be SCFA producers, especially acetate, propionate, and butyric acids [93,94,95]. The RS fraction of OS starch may be a preferred substrate for these bacterial genera, thus promoting their growth. The increased abundance of *Alistipes* reduces the risk of inflammatory bowel disease [96] and *Clostridium difficile* infection [97]. *Parabacteroides* were proposed to suppress inflammatory responses and are negatively associated with weight, blood pressure, blood lipids, and glucose levels [98,99]. The relative abundance of *Ruminiclostridium_5* genera was also increased by OS starch intervention, but the physiological effects of this genus have not been thoroughly investigated.

OS starch might play a critical role in the inhibition of pathogenic bacteria. *Desulfovibrio*, *Oscillibacter*, *Tyzzerella*, *Anaerotruncus*, and *Lachnospiraceae_UCG-006* were significantly decreased by OS starch intervention compared to those in the HF group. These bacteria were reported to be positively associated with the consumption of an HF diet [15,90,91,100]. *Desulfovibrio*, which belongs to the phylum *Proteobacteria*, was shown to increase in ulcerative colitis patients [101,102]. *Desulfovibrio* may promote inflammation in the colon [15]. *Oscillibacter* is a potentially harmful bacterium with proinflammatory effects [103] and was shown to be positively associated with inflammatory bowel disease [104], colorectal cancer [105], and ulcerative colitis [106]. *Anaerotruncus* and *Tyzzerella* were found to be promoted by a high-saturated fat and low-fiber diet, which could be related to the development of some disorders, such as obesity, cardiovascular disease, and other proinflammatory diseases [107,108,109]. In addition, the relative abundances of *Bacteroides* and *Alloprevotella* in the HFOSS group were the same as those in the RF group and were increased compared to those in the HF group, but the difference was not significant (*p* > 0.05). Both *Bacteroides* and *Alloprevotella* were negatively correlated with body weight, which is consistent with our results [110,111,112].

#### 3.6.3. Community Richness and Diversity of Fecal Microbiota

**Figure 8:**
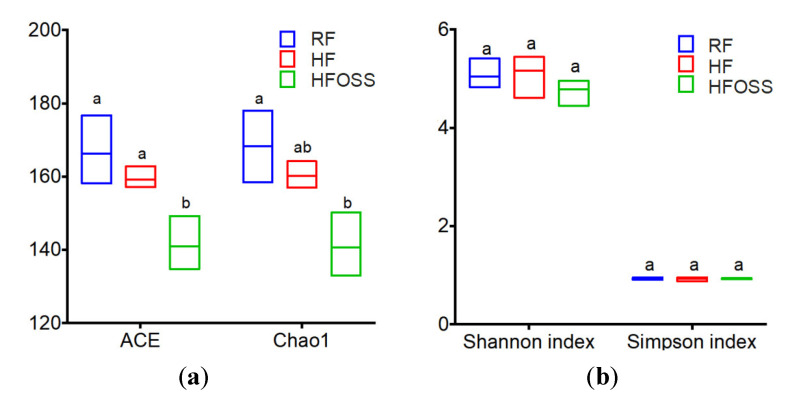
Boxplots of between-group α-diversity comparison. (**a**) ACE index and Chao 1 index boxplots as well as (**b**) Shannon index and Simpson index boxplots of the regular-fat diet group (RF), high-fat diet group (HF), and high-fat diet supplemented with OS starch group (HFOSS). Each box diagram shows the minimum, first quartile, medium, third quartile, and maximum values of the index of the corresponding group. Different letters indicate significant differences between groups (*p* < 0.05).

Alpha-diversity analysis was performed to assess the diversity of fecal microbiota (Figure 8a,b). The HF and RF groups had similar α-diversity indices, suggesting that the fecal microbiota in these two groups had a similar abundance and diversity of species. The ACE index of the HFOSS group were significantly lower than that of the HF group. No significant differences in the Shannon and Simpson diversity indices were observed among the three groups.

ACE and Chao 1 indices were used to assess the total number of species, while Shannon and Simpson’s indices reflect the diversity of microbials. It is interesting to notice that the HFOSS group has fewer species than RF and HF groups. Supplementing resistant starch to an HF diet is not always correlated with more species in the gut [113]; this may attribute to the structure and type of the resistant starch [114]. Some bacteria may be vanished due to the lack of substrates; in addition, the gut environment created by OS starch supplementation may not be suitable for the growth of some bacteria. For example, *Escherichia-Shigella*, [*Eubacterium*]_*fissicatena_group*, and *Defluviitaleaceae_UCG-011* were found in RF and HF groups but did not exist in the HFOSS group. It is also worth mentioning that cellulose was replaced by OS starch in the HFOSS group, which may affect the gut microbial diversity. Whether the metabolites of cellulose and OS starch have an across-feeding effect on bacteria is less investigated, which can be a subject for investigation in the future.

**Figure 9:**
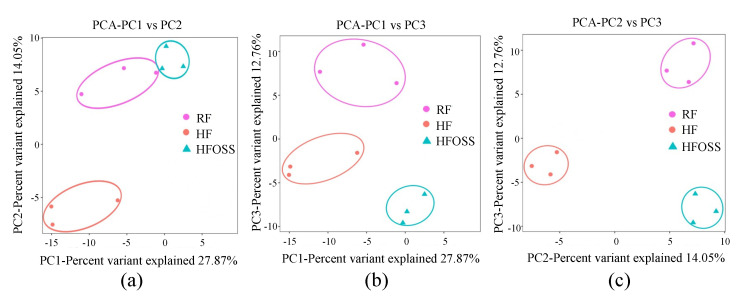
Principle components analysis (PCA) results. (**a**) PC1 vs. PC2, (**b**) PC1 vs. PC3, and (**c**) PC2 vs. PC3. Each point in the figure represents a sample. The distance between points represents the variance of different bacterial communities of the sample. The closer the distance between the points, the higher the similarity between the samples.

Principal component analysis (PCA) showed that samples from the same group clustered together but were further away from samples in the other groups (Figure 9). A similar result was obtained by Anosim analysis, indicating that the difference between groups was greater than the differences within groups (Figure S3). The results suggest that OS starch intervention significantly changed the fecal microbiota structure of mice fed an HF diet.

## 4. Conclusions

OS starch was found to prevent body fat accumulation and improve glucose and insulin tolerance in mice fed an HF diet. The body composition of the HFOSS group was similar to the RF group. OS starch suppressed the appetite of the HF diet-fed mice and increased their fecal fat secretion, which may be beneficial for body weight control. The OS starch contains a significant fraction of resistant starch, which can enter the large intestine and be fermented by gut microbes. OS starch increases fecal SCFA concentrations, especially acetic, propionic, and butyric acid, which may play a critical role in maintaining metabolic health in subjects. OS starch intervention significantly altered the fecal microbiota structure of mice fed an HF diet. The F/B ratio of mice fed an HF diet was significantly decreased by OS starch intervention, which was at a similar level as the RF group. The relative abundances of *Proteobacteria* and *Deferribacteres* in the HFOSS group were lower than those in the HF group. At the genus level, OS starch increased the relative abundance of probiotics such as *Parabacteroides* and *Alistipes* and decreased the relative abundance of pathogenic bacteria including *Tyzzerella*, *Oscillibacter*, *Desulfovibrio*, *Anaerotruncus*, and *Lachnospiraceae_UCG-006* in the feces of mice fed an HF diet. OS starch intervention decreased community richness but did not change the microbial diversity indices of the fecal microbiota of HF diet-fed mice. In summary, OS starch appears to have some nutritional functions. Its health benefits may be due to its fermentability in the gut and its ability to regulate the structure of the gut microbial community.

## Figures and Tables

**Table 1:** Components of diets from weeks 10 to 22.

Ingredient	Regular-Fat (RF) Diet (g)	High-Fat (HF) Diet (g)	High-Fat Diet Supplemented with OS Starch (HFOSS) ^1^ (g)
Casein	200.0	200.0	200.0
Sucrose	68.8	68.8	56.2
Corn starch	506.2	0.0	0.0
Maltodextrin	125.0	125.0	0.0
Soy oil	25.0	25.0	25.0
Lard	20.0	245.0	245.0
Cellulose	50.0	50.0	0.0
OS starch ^1^	0.0	0.0	215.0
Mineral Mix S10026	10.0	10.0	10.0
Dicalcium phosphate	13.0	13.0	13.0
Calcium carbonate	5.5	5.5	5.5
Potassium citrate	16.5	16.5	16.5
Vitamin mix V10001	10.0	10.0	10.0
Choline bitartrate	2.0	2.0	2.0
L-Cysteine	3.0	3.0	3.0
Ethoxyquin	0.035	0.035	0.035
Energy density (kcal/g)	3.8	5.2	5.1
Percentage of energy provided by macronutrients (%)			
Fat	10.1	60.5	60.6
Carbohydrate	69.7	19.3	19.1
Protein	20.2	20.2	20.3

^1^ OS stands for octenylsuccinate.

## Data Availability

The data presented in this study are available on request from the corresponding author. The data are not publicly available due to privacy.

## Supplementary Materials

The following supporting information can be downloaded at: https://www.mdpi.com/article/10.3390/foods11192980/s1, Figure S1: Plasma GLP-1 level of mice fed a regular-fat diet (RF), high-fat diet (HF) and high-fat diet supplemented with OS starch (HFOSS). Values with different lowercase letters indicate significant differences between groups (*p* < 0.05); Figure S2: Differential analysis of genus composition between the high-fat diet (HF) group and high-fat diet supplemented with OS starch (HFOSS) group. Each box diagram shows the minimum, first quartile, medium, third quartile and maximum values of the index of the corresponding group; Figure S3: Group comparison by Anosim analysis. (a) Anosim analysis of mouse fecal microbiota between the high-fat diet (HF) group and regular-fat diet (RF) group. (b) Anosim analysis of mouse fecal microbiota between the HF group and the high-fat diet supplemented with OS starch (HFOSS) group; Table S1: Components of diets from week 1 to 9; Section S2: Methods of histopathological examination.

## Abbreviations

AUC, area under the curve; AMPK, AMP-activated protein kinase; DS, degree of substitution; F/B, the ratio of *Firmicutes* to *Bacteroidetes*; FID, flame ionization detector; GC, gas chromatography; GLP-1, glucagon-like peptide 1; H&E, hematoxylin–eosin; HF, high fat; HFOSS, high-fat diet supplemented with OS starch; IPGTT, intraperitoneal glucose tolerance tests; ITT, insulin tolerance test; OS, octenylsuccinate; OSA, octenylsuccinic anhydride; OTUs, operational taxonomic units; PPAR, peroxisome proliferator-activated receptor; PYY, peptide YY; RDS, rapidly digestible starch; RE, reaction efficiency; RF, regular fat; RS, resistant starch; SCFAs, short-chain fatty acids; SD, standard deviation; SDS, slowly digestible starch.

## References

[1] Bai Y., Shi Y.-C. (2011). Structure and preparation of octenyl succinic esters of granular starch, microporous starch and soluble maltodextrin. Carbohydr. Polym..

[2] Sweedman M.C., Tizzotti M.J., Schäfer C., Gilbert R.G. (2013). Structure and physicochemical properties of octenyl succinic anhydride modified starches: A review. Carbohydr. Polym..

[3] Caldwell C.G., Wurzburg O.B. (1953). Polysaccharide Derivatives of Substituted Dicarboxylic Acids. U.S. Patent.

[4] Tesch S., Gerhards C., Schubert H. (2002). Stabilization of emulsions by OSA starches. J. Food Eng..

[5] Altuna L., Herrera M.L., Foresti M.L. (2018). Synthesis and characterization of octenyl succinic anhydride modified starches for food applications. A review of recent literature. Food Hydrocoll..

[6] Hategekimana J., Masamba K.G., Ma J., Zhong F. (2015). Encapsulation of vitamin E: Effect of physicochemical properties of wall material on retention and stability. Carbohydr. Polym..

[7] Wolf B.W., Wolever T.M.S., Bolognesi C., Zinker B.A., Garleb K.A., Firkins J.L. (2001). Glycemic Response to a food starch esterified by 1-octenyl succinic anhydride in humans. J. Agric. Food Chem..

[8] He J., Liu J., Zhang G. (2008). Slowly digestible waxy maize starch prepared by octenyl succinic anhydride esterification and heat−moisture treatment: Glycemic response and mechanism. Biomacromolecules.

[9] Heacock P.M., Hertzler S.R., Wolf B. (2004). The glycemic, insulinemic, and breath hydrogen responses in humans to a food starch esterified by 1-octenyl succinic anhydride. Nutr. Res..

[10] Huang J., Chang R., Ma R., Zhan J., Lu X., Tian Y. (2022). Effects of structure and physical chemistry of resistant starch on short-term satiety. Food Hydrocoll..

[11] Power S.E., O’Toole P.W., Stanton C., Ross R.P., Fitzgerald G.F. (2013). Intestinal microbiota, diet and health. Br. J. Nutr..

[12] Rogero M.M., Calder P.C. (2018). Obesity, inflammation, toll-Like receptor 4 and fatty Acids. Nutrients.

[13] Araújo J.R., Tomas J., Brenner C., Sansonetti P.J. (2017). Impact of high-fat diet on the intestinal microbiota and small intestinal physiology before and after the onset of obesity. Biochimie.

[14] Lee K.Y., Yoo S.-H., Lee H.G. (2012). The effect of chemically-modified resistant starch, RS type-4, on body weight and blood lipid profiles of high fat diet-induced obese mice. Starch-Starke.

[15] Zhang Y., Chen L., Hu M., Kim J.J., Lin R., Xu J., Fan L., Qi Y., Wang L., Liu W. (2020). Dietary type 2 resistant starch improves systemic inflammation and intestinal permeability by modulating microbiota and metabolites in aged mice on high-fat diet. Aging.

[16] Si X., Strappe P., Blanchard C., Zhou Z. (2017). Enhanced anti-obesity effects of complex of resistant starch and chitosan in high fat diet fed rats. Carbohydr. Polym..

[17] Rosado C.P., Rosa V.H.C., Martins B.C., Soares A.C., Santos I.B., Monteiro E.B., Moura-Nunes N., da Costa C.A., Mulder A.D.R.P., Daleprane J.B. (2020). Resistant starch from green banana (*Musa sp.*) attenuates non-alcoholic fat liver accumulation and increases short-chain fatty acids production in high-fat diet-induced obesity in mice. Int. J. Biol. Macromol..

[18] Polakof S., Díaz-Rubio M.E., Dardevet D., Martin J.-F., Pujos-Guillot E., Scalbert A., Sebedio J.-L., Mazur A., Comte B. (2013). Resistant starch intake partly restores metabolic and inflammatory alterations in the liver of high-fat-diet-fed rats. J. Nutr. Biochem..

[19] Xu J., Ma Z., Li X., Liu L., Hu X. (2020). A more pronounced effect of type III resistant starch *vs.* type II resistant starch on ameliorating hyperlipidemia in high fat diet-fed mice is associated with its supramolecular structural characteristics. Food Funct..

[20] Shen R.-L., Zhang W.-L., Dong J.-L., Ren G.-X., Chen M. (2015). Sorghum resistant starch reduces adiposity in high-fat diet-induced overweight and obese rats via mechanisms involving adipokines and intestinal flora. Food Agric. Immunol..

[21] Fu J., Wang Y., Tan S., Wang J. (2021). Effects of banana resistant starch on the biochemical indexes and intestinal flora of obese rats induced by a high-fat diet and their correlation analysis. Front. Bioeng. Biotechnol..

[22] Zaman S.A., Sarbini S.R. (2016). The potential of resistant starch as a prebiotic. Crit. Rev. Biotechnol..

[23] Fuentes-Zaragoza E., Sánchez-Zapata E., Sendra E., Sayas E., Navarro C., Fernández-López J., Pérez-Alvarez J.A. (2011). Resistant starch as prebiotic: A review. Starch-Stärke.

[24] Shang W., Si X., Zhou Z., Li Y., Strappe P., Blanchard C. (2017). Characterization of fecal fat composition and gut derived fecal microbiota in high-fat diet fed rats following intervention with chito-oligosaccharide and resistant starch complexes. Food Funct..

[25] Shou D., Cao C., Xu H., Huang H., Xia Y., Mei Q., Quan Y., Chen H., Zhao C., Tang W. (2021). Type 2 resistant starch improves liver steatosis induced by high-fat diet relating to gut microbiota regulation and concentration of propionic acid in portal vein blood in C57BL/6J mice. Gut.

[26] Zhou Y., Zhao S., Jiang Y., Wei Y., Zhou X. (2019). Regulatory function of buckwheat-resistant starch supplementation on lipid profile and gut microbiota in mice fed with a high-fat diet. J. Food Sci..

[27] Ai Y., Nelson B., Birt D., Jane J.-L. (2013). *In vitro* and *in vivo* digestion of octenyl succinic starch. Carbohydr. Polym..

[28] Zhang B., Mei J.-Q., Chen B., Chen H.-Q. (2017). Digestibility, physicochemical and structural properties of octenyl succinic anhydride-modified cassava starches with different degree of substitution. Food Chem..

[29] Wolf B.W., Bauer L.L., Fahey G.C. (1999). Effects of chemical modification on *in vitro* rate and extent of food starch digestion: An attempt to discover a slowly digested starch. J. Agric. Food Chem..

[30] Han J.-A., BeMiller J.N. (2007). Preparation and physical characteristics of slowly digesting modified food starches. Carbohydr. Polym..

[31] Liang S., Hong Y., Gu Z., Cheng L., Li C., Li Z. (2021). Effect of debranching on the structure and digestibility of octenyl succinic anhydride starch nanoparticles. LWT.

[32] Olawoye B., Fagbohun O.F., Popoola O.O., Gbadamosi S.O., Akanbi C.T. (2022). Understanding how different modification processes affect the physiochemical, functional, thermal, morphological structures and digestibility of cardaba banana starch. Int. J. Biol. Macromol..

[33] Remya R., Jyothi A.N., Sreekumar J. (2017). Comparative study of RS4 type resistant starches derived from cassava and potato starches via octenyl succinylation. Starch-Stärke.

[34] No J., Mun S., Shin M. (2019). Properties and digestibility of octenyl succinic anhydride-modified Japonica-type waxy and non-waxy rice starches. Molecules.

[35] Lan X., Huang B., Wu J., Wang Z. (2015). The effect of octenylsuccinylation on morphological, rheological, and *in vitro* digestibility properties of *Canna edulis* Ker starch. Starch-Stärke.

[36] Quintero-Castaño V.D., Castellanos-Galeano F.J., Álvarez-Barreto C.I., Bello-Pérez L.A., Alvarez-Ramirez J. (2020). *In vitro* digestibility of octenyl succinic anhydride-starch from the fruit of three Colombian Musa. Food Hydrocoll..

[37] Lu K., Miao M., Ye F., Cui S.W., Li X., Jiang B. (2016). Impact of dual-enzyme treatment on the octenylsuccinic anhydride esterification of soluble starch nanoparticle. Carbohydr. Polym..

[38] Simsek S., Ovando-Martínez M., Marefati A., Sjöö M., Rayner M. (2015). Chemical composition, digestibility and emulsification properties of octenyl succinic esters of various starches. Food Res. Int..

[39] Sharma M., Singh A.K., Yadav D.N., Arora S., Vishwakarma R. (2016). Impact of octenyl succinylation on rheological, pasting, thermal and physicochemical properties of pearl millet (*Pennisetum typhoides*) starch. LWT.

[40] Zheng Y., Hu L., Ding N., Liu P., Yao C., Zhang H. (2017). Physicochemical and structural characteristics of the octenyl succinic ester of ginkgo starch. Int. J. Biol. Macromol..

[41] Lv Q.-Q., Li G.-Y., Xie Q.-T., Zhang B., Li X.-M., Pan Y., Chen H.-Q. (2018). Evaluation studies on the combined effect of hydrothermal treatment and octenyl succinylation on the physic-chemical, structural and digestibility characteristics of sweet potato starch. Food Chem..

[42] Ferguson M.J., Jones G.P. (2000). Production of short-chain fatty acids following *in vitro* fermentation of saccharides, saccharide esters, fructo-oligosaccharides, starches, modified starches and non-starch polysaccharides. J. Sci. Food Agric..

[43] Shi Y.C., Bai Y. (2016). Starch esters and method of preparation. U.S. Patent.

[44] Englyst K.N., Englyst H.N., Hudson G.J., Cole T.J., Cummings J.H. (1999). Rapidly available glucose in foods: An *in vitro* measurement that reflects the glycemic response. Am. J. Clin. Nutr..

[45] Sang Y., Seib P.A. (2006). Resistant starches from amylose mutants of corn by simultaneous heat-moisture treatment and phosphorylation. Carbohydr. Polym..

[46] Chen J., Xiao J., Wang Z., Cheng H., Zhang Y., Lin B., Qin L., Bai Y. (2020). Effects of reaction condition on glycosidic linkage structure, physical–chemical properties and *in vitro* digestibility of pyrodextrins prepared from native waxy maize starch. Food Chem..

[47] Wolever T.M., Jenkins D.J., Jenkins A.L., Josse R.G. (1991). The glycemic index: Methodology and clinical implications. Am. J. Clin. Nutr..

[48] Kritchevsky D., Tepper S.A. (2005). Influence of a fiber mixture on serum and liver lipids and on fecal fat excretion in rats. Nutr. Res..

[49] García-Villalba R., Giménez-Bastida J.A., García-Conesa M.T., Tomás-Barberán F.A., Carlos Espín J., Larrosa M. (2012). Alternative method for gas chromatography-mass spectrometry analysis of short-chain fatty acids in faecal samples. J. Sep. Sci..

[50] Deming L., Qian H., Huanhuan Y., Yafang D., Kangqing Y., Jing Y., Xing T., Yaxin G., Jiaying X., Liqiang Q. (2021). Daily dose of bovine lactoferrin prevents ethanol-induced liver injury and death in male mice by regulating hepatic alcohol metabolism and modulating gut microbiota. Mol. Nutr. Food Res..

[51] Liu F., Wang X., Li D., Cui Y., Li X. (2021). Apple polyphenols extract alleviated dextran sulfate sodium-induced ulcerative colitis in C57BL/6 male mice by restoring bile acid metabolism disorder and gut microbiota dysbiosis. Phytother. Res..

[52] Birt D.F., Boylston T., Hendrich S., Jane J.-L., Hollis J., Li L., McClelland J., Moore S., Phillips G.J., Rowling M. (2013). Resistant starch: Promise for improving human health. Adv. Nutr. Int. Rev. J..

[53] Keenan M.J., Zhou J., Hegsted M., Pelkman C., Durham H.A., Coulon D.B., Martin R.J. (2015). Role of resistant starch in improving gut health, adiposity, and insulin resistance. Adv. Nutr..

[54] Aziz A.A., Kenney L.S., Goulet B., Abdel-Aal E.-S. (2009). Dietary starch type affects body weight and glycemic control in freely fed but not energy-restricted obese rats. J. Nutr..

[55] Shen L., Keenan M.J., Martin R.J., Tulley R.T., Raggio A.M., McCutcheon K.L., Zhou J. (2009). Dietary resistant starch increases hypothalamic POMC expression in rats. Obesity.

[56] Zhou J., Martin R.J., Tulley R.T., Raggio A.M., McCutcheon K.L., Shen L., Danna S.C., Tripathy S., Hegsted M., Keenan M.J. (2008). Dietary resistant starch upregulates total GLP-1 and PYY in a sustained day-long manner through fermentation in rodents. Am. J. Physiol. Metab..

[57] Keenan M.J., Martin R.J., Raggio A.M., McCutcheon K.L., Brown I.L., Birkett A., Newman S.S., Skaf J., Hegsted M., Tulley R.T. (2012). High-Amylose resistant starch increases hormones and improves structure and function of the gastrointestinal tract: A microarray study. Lifestyle Genom..

[58] Zhou J., Martin R.J., Raggio A.M., Shen L., McCutcheon K., Keenan M.J. (2015). The importance of GLP-1 and PYY in resistant starch’s effect on body fat in mice. Mol. Nutr. Food Res..

[59] Lehmann U., Robin F. (2007). Slowly digestible starch - its structure and health implications: A review. Trends Food Sci. Technol..

[60] Koh A., De Vadder F., Kovatcheva-Datchary P., Bäckhed F. (2016). From dietary fiber to host physiology: Short-chain fatty acids as key bacterial metabolites. Cell.

[61] Li M., Wang J., Wang F., Strappe P., Liu W., Zheng J., Zhou Z., Zhang Y. (2021). Microbiota fermentation characteristics of acylated starches and the regulation mechanism of short-chain fatty acids on hepatic steatosis. Food Funct..

[62] Jiminez J.A., Uwiera T.C., Abbott D.W., Uwiera R.R.E., Inglis G.D. (2016). Impacts of resistant starch and wheat bran consumption on enteric inflammation in relation to colonic bacterial community structures and short-chain fatty acid concentrations in mice. Gut Pathog..

[63] Chassaing B., Koren O., Goodrich J.K., Poole A.C., Srinivasan S., Ley R.E., Gewirtz A.T. (2015). Dietary emulsifiers impact the mouse gut microbiota promoting colitis and metabolic syndrome. Nature.

[64] Topping D.L., Clifton P.M. (2001). Short-chain fatty acids and human colonic function: Roles of resistant starch and nonstarch polysaccharides. Physiol. Rev..

[65] Belobrajdic D.P., King R.A., Christophersen C.T., Bird A.R. (2012). Dietary resistant starch dose-dependently reduces adiposity in obesity-prone and obesity-resistant male rats. Nutr. Metab..

[66] Wan J., Wu Y., Pham Q., Yu L. (2020). Effects of rice with different amounts of resistant starch on mice fed a high-fat diet: Attenuation of adipose weight gain. J. Agric. Food Chem..

[67] Liang D., Zhang L., Chen H., Zhang H., Hu H., Dai X. (2021). Potato resistant starch inhibits diet-induced obesity by modifying the composition of intestinal microbiota and their metabolites in obese mice. Int. J. Biol. Macromol..

[68] Chang D., Hu X., Ma Z. (2022). Pea-Resistant starch with different multi-scale structural features attenuates the obesity-related physiological changes in high-fat diet mice. J. Agric. Food Chem..

[69] Van der Beek C.M., Canfora E.E., Lenaerts K., Troost F.J., Damink S.W.M.O., Holst J.J., Masclee A.A.M., Dejong C.H.C., Blaak E.E. (2016). Distal, not proximal, colonic acetate infusions promote fat oxidation and improve metabolic markers in overweight/obese men. Clin. Sci..

[70] Besten G.d., Bleeker A., Gerding A., Eunen K.v., Havinga R., Dijk T.H.v., Oosterveer M.H., Jonker J.W., Groen A.K., Reijngoud D.-J. (2015). Short-chain fatty acids protect against high-fat diet-induced obesity via a PPARγ-dependent switch from lipogenesis to fat oxidation. Diabetes.

[71] Lu Y., Fan C., Li P., Lu Y., Chang X., Qi K. (2016). Short chain fatty acids prevent high-fat-diet-induced obesity in mice by regulating g protein-coupled receptors and gut microbiota. Sci. Rep..

[72] Frost G., Sleeth M.L., Sahuri-Arisoylu M., Lizarbe B., Cerdan S., Brody L., Anastasovska J., Ghourab S., Hankir M., Zhang S. (2014). The short-chain fatty acid acetate reduces appetite via a central homeostatic mechanism. Nat. Commun..

[73] Portincasa P., Bonfrate L., Vacca M., De Angelis M., Farella I., Lanza E., Khalil M., Wang D.Q.-H., Sperandio M., Di Ciaula A. (2022). Gut microbiota and short chain fatty acids: Implications in glucose homeostasis. Int. J. Mol. Sci..

[74] Chakraborti C.K. (2015). New-found link between microbiota and obesity. World J. Gastrointest. Pathophysiol..

[75] Lim S., Choo J., Li H., O’Rielly R., Carragher J., Rogers G., Searle I., Robertson S., Page A., Muhlhausler B. (2021). A high amylose wheat diet improves gastrointestinal health parameters and gut microbiota in male and female mice. Foods.

[76] Martínez I., Kim J., Duffy P.R., Schlegel V.L., Walter J. (2010). Resistant starches types 2 and 4 have differential effects on the composition of the fecal microbiota in human subjects. PLoS ONE.

[77] Herrmann E., Young W., Rosendale D., Conrad R., Riedel C.U., Egert M. (2017). Determination of resistant starch assimilating bacteria in fecal samples of mice by *in vitro* RNA-based stable isotope probing. Front. Microbiol..

[78] Ley R.E., Bäckhed F., Turnbaugh P., Lozupone C.A., Knight R.D., Gordon J.I. (2005). Obesity alters gut microbial ecology. Proc. Natl. Acad. Sci. USA.

[79] Ley R.E., Turnbaugh P.J., Klein S., Gordon J.I. (2006). Microbial ecology: Human gut microbes associated with obesity. Nature.

[80] Jo J.-K., Seo S.-H., Park S.-E., Kim H.-W., Kim E.-J., Kim J.-S., Pyo J.-Y., Cho K.-M., Kwon S.-J., Park D.-H. (2021). Gut microbiome and metabolome profiles associated with high-fat diet in mice. Metabolites.

[81] Wang B., Yu H., He Y., Wen L., Gu J., Wang X., Miao X., Qiu G., Wang H. (2021). Effect of soybean insoluble dietary fiber on prevention of obesity in high-fat diet fed mice *via* regulation of the gut microbiota. Food Funct..

[82] Cai H., Wen Z., Li X., Meng K., Yang P. (2020). *Lactobacillus plantarum* FRT10 alleviated high-fat diet–induced obesity in mice through regulating the PPARα signal pathway and gut microbiota. Appl. Microbiol. Biotechnol..

[83] Murphy E.F., Cotter P.D., Healy S., Marques T.M., O’Sullivan O., Fouhy F., Clarke S.F., O’Toole P.W., Quigley E.M., Stanton C. (2010). Composition and energy harvesting capacity of the gut microbiota: Relationship to diet, obesity and time in mouse models. Gut.

[84] Li Z.-T., Hu G.-A., Zhu L., Zhao Z.-C., Jiang Y., Gao M.-J., Zhan X.-B. (2021). *In vitro* digestion and fecal fermentation of highly resistant starch rice and its effect on the gut microbiota. Food Chem..

[85] Maharshak N., Packey C.D., Ellermann M., Manick S., Siddle J.P., Huh E.Y., Plevy S.E., Sartor R.B., Carroll I.M. (2013). Altered enteric microbiota ecology in interleukin 10-deficient mice during development and progression of intestinal inflammation. Gut Microbes.

[86] Shin N.-R., Whon T.W., Bae J.-W. (2015). *Proteobacteria*: Microbial signature of dysbiosis in gut microbiota. Trends Biotechnol..

[87] Zhang C., Zhang M., Pang X., Zhao Y., Wang L., Zhao L. (2012). Structural resilience of the gut microbiota in adult mice under high-fat dietary perturbations. ISME J..

[88] Liu Y., Li T., Alim A., Ren D., Zhao Y., Yang X. (2019). Regulatory effects of stachyose on colonic and hepatic inflammation, gut microbiota dysbiosis, and peripheral CD4+ T cell distribution abnormality in high-fat diet-fed mice. J. Agric. Food Chem..

[89] Li X., Wang Y., Xing Y., Xing R., Liu Y., Xu Y. (2020). Changes of gut microbiota during silybin-mediated treatment of high-fat diet-induced non-alcoholic fatty liver disease in mice. Hepatol. Res..

[90] Vacca M., Celano G., Calabrese F.M., Portincasa P., Gobbetti M., De Angelis M. (2020). The controversial role of human gut *Lachnospiraceae*. Microorganisms.

[91] Rainey F.A., Family V., De Vos P., Garrity G.M., Jones D., Krieg N.R., Ludwig W., Rainey F.A., Schleifer K.-H., Whitman W.B. (2009). *Lachnospiraceae* fam. nov. Bergey’s Manual of Systematic Bacteriology.

[92] Guo M., Li Z. (2019). Polysaccharides isolated from *Nostoc commune* Vaucher inhibit colitis-associated colon tumorigenesis in mice and modulate gut microbiota. Food Funct..

[93] Yang D., Lyu W., Hu Z., Gao J., Zheng Z., Wang W., Firrman J., Ren D. (2021). Probiotic effects of *lactobacillus fermentum zjuids06* and *lactobacillus plantarum zy08* on hypercholesteremic golden hamsters. Front. Nutr..

[94] Hu Y., Le Leu R.K., Christophersen C.T., Somashekar R., Conlon M.A., Meng X.Q., Winter J.M., Woodman R.J., McKinnon R., Young G.P. (2016). Manipulation of the gut microbiota using resistant starch is associated with protection against colitis-associated colorectal cancer in rats. Carcinogenesis.

[95] Parker B.J., Wearsch P.A., Veloo A.C.M., Rodriguez-Palacios A. (2020). The genus *Alistipes*: Gut bacteria with emerging implications to inflammation, Cancer, and Mental Health. Front. Immunol..

[96] Dziarski R., Park S.Y., Kashyap D., Dowd S., Gupta D. (2016). Pglyrp-regulated gut microflora *Prevotella falsenii, Parabacteroides distasonis* and *Bacteroides eggerthii* Enhance and *Alistipes finegoldii* attenuates colitis in mice. PLoS ONE.

[97] Schubert A.M., Sinani H., Schloss P.D. (2015). Antibiotic-induced alterations of the murine gut microbiota and subsequent effects on colonization resistance against *Clostridium difficile*. mBio.

[98] Arpaia N., Campbell C., Fan X., Dikiy S., Van Der Veeken J., DeRoos P., Liu H., Cross J.R., Pfeffer K., Coffer P.J. (2013). Metabolites produced by commensal bacteria promote peripheral regulatory T-cell generation. Nature.

[99] Zeng Q., Li D., He Y., Li Y., Yang Z., Zhao X., Liu Y., Wang Y., Sun J., Feng X. (2019). Discrepant gut microbiota markers for the classification of obesity-related metabolic abnormalities. Sci. Rep..

[100] Kong C., Gao R., Yan X., Huang L., Qin H. (2019). Probiotics improve gut microbiota dysbiosis in obese mice fed a high-fat or high-sucrose diet. Nutrition.

[101] Lennon G., Balfe Á., Bambury N., Lavelle A., Maguire A., Docherty N.G., Coffey J.C., Winter D.C., Sheahan K., O’Connell P.R. (2014). Correlations between colonic crypt mucin chemotype, inflammatory grade and *Desulfovibrio* species in ulcerative colitis. Colorectal Dis..

[102] Rowan F., Docherty N.G., Murphy M., Murphy B., Coffey J.C., O’Connell P.R. (2010). *Desulfovibrio* bacterial species are increased in ulcerative colitis. Dis. Colon Rectum.

[103] Li Y., Liu M., Liu H., Sui X., Liu Y., Wei X., Liu C., Cheng Y., Ye W., Gao B. (2021). The anti-inflammatory effect and mucosal barrier protection of *Clostridium butyricum* RH2 in ceftriaxone-induced intestinal dysbacteriosis. Front. Cell. Infect. Microbiol..

[104] Peng Y., Yan Y., Wan P., Chen D., Ding Y., Ran L., Mi J., Lu L., Zhang Z., Li X. (2019). Gut microbiota modulation and anti-inflammatory properties of anthocyanins from the fruits of *Lycium ruthenicum* Murray in dextran sodium sulfate-induced colitis in mice. Free Radic. Biol. Med..

[105] Wang C.-S., Li W.-B., Wang H.-Y., Ma Y.-M., Zhao X.-H., Yang H., Qian J.-M., Li J.-N. (2018). VSL#3 can prevent ulcerative colitis-associated carcinogenesis in mice. World J. Gastroenterol..

[106] Wu M., Li P., An Y., Ren J., Yan D., Cui J., Li D., Li M., Wang M., Zhong G. (2019). Phloretin ameliorates dextran sulfate sodium-induced ulcerative colitis in mice by regulating the gut microbiota. Pharmacol. Res..

[107] Bailén M., Bressa C., Martínez-López S., González-Soltero R., Lominchar M.G.M., Juan C.S., Larrosa M. (2020). Microbiota features associated with a high-fat/low-fiber diet in healthy adults. Front. Nutr..

[108] Kelly T.N., Bazzano L.A., Ajami N.J., He H., Zhao J., Petrosino J.F., Correa A., He J. (2016). Gut microbiome associates with lifetime cardiovascular disease risk profile among bogalusa heart study participants. Circ. Res..

[109] Fernandez D.M., Clemente J.C., Giannarelli C. (2018). Physical activity, immune system, and the microbiome in cardiovascular disease. Front. Physiol..

[110] Guo W.-L., Pan Y.-Y., Li L., Li T.-T., Liu B., Lv X.-C. (2018). Ethanol extract of *Ganoderma lucidum* ameliorates lipid metabolic disorders and modulates the gut microbiota composition in high-fat diet fed rats. Food Funct..

[111] Li T.-T., Huang Z.-R., Jia R.-B., Lv X.-C., Zhao C., Liu B. (2021). *Spirulina platensis* polysaccharides attenuate lipid and carbohydrate metabolism disorder in high-sucrose and high-fat diet-fed rats in association with intestinal microbiota. Food Res. Int..

[112] Wan X., Li T., Liu D., Chen Y., Liu Y., Liu B., Zhang H., Zhao C. (2018). Effect of marine microalga *Chlorella pyrenoidosa* ethanol extract on lipid metabolism and gut microbiota composition in high-fat diet-fed rats. Mar. Drugs.

[113] Zhu Y., Dong L., Huang L., Shi Z., Dong J., Yao Y., Shen R. (2020). Effects of oat β-glucan, oat resistant starch, and the whole oat flour on insulin resistance, inflammation, and gut microbiota in high-fat-diet-induced type 2 diabetic rats. J. Funct. Foods.

[114] Flint H.J., Shi Y.-C., Maningat C.C. (2013). The microbiology of resistant starch fermentation in the human large intestine: A host of unanswered questions. Resistant Starch.

