# Supplementary material for: Octenyl Succinic Anhydride-Modified Starch Attenuates Body Weight Gain and Changes Intestinal Environment of High-Fat Diet-Fed Mice
Source: Foods. 2022 Sep 23;11(19):2980. doi: 10.3390/foods11192980 (PMC9563757; doi:10.3390/foods11192980)
Supplement: Supplementary file 1 [file foods-11-02980-s001.zip › foods-1818365-supplementary.pdf]

Supplementary Materials:

Table S1. Components of Diets from Week 1 to 9.

Ingredient	Regular-fat diet (g)	High-fat diet (g)	High-fat diet supplemented with OS starch[1] (g)
Casein	200.0	200.0	200.0
Sucrose	350.0	172.8	172.8
Corn starch	315.0	72.8	0.0
Maltodextrin	35.0	100.0	25.1
Soy oil	25.0	25.0	25.0
Lard	20.0	177.5	177.5
Cellulose	50.0	50.0	0.0
OS starch[1]	0.0	0.0	240.0
Mineral Mix S10026	10.0	10.0	10.0
Dicalcium phosphate	13.0	13.0	13.0
Calcium carbonate	5.5	5.5	5.5
Potassium citrate	16.5	16.5	16.5
Vitamin mix V10001	10.0	10.0	10.0
Choline bitartrate	2.0	2.0	2.0
L-Cysteine	3.0	3.0	3.0
Ethoxyquin	0.008	0.008	0.008
Percentage of energy provided by macronutrients (%)			
Fat	10.1	45.5	45.5
Carbohydrate	69.9	34.5	34.5
Protein	20.0	20.0	20.0
Energy density (kcal/g)	3.8	4.7	4.3

[1] OS stands for octenylsuccinate.

S2. Methods of Histopathological Examination

S2.1. Sample Preparation and H&E Staining

The tissue was removed from the 10% neutral formalin solution, trimmed and placed with a label in a dehydration box. The tissue was dehydrated with gradient alcohol followed by wax leaching. The wax-soaked tissue was embedded in the embedding machine and cooled on a -20 °C freezing table. The wax block was sliced on a paraffin slicer to a thickness of 4 μm. The flattened tissue was picked up by a glass slide and baked in an oven at 60 °C. After the wax is melted, the slide was taken out and stored at room temperature for further treatment. Before staining, the tissue was dewaxed following a standard method. The section was stained with Hematoxylin solution, Hematoxylin Differentiation solution, and Hematoxylin Scott Tap Bluing following a standard procedure. Then the section was treated with ethanol and stained with Eosin dye. The slide was dehydrated and sealed with a neutral gum.

S2.2. Sample Preparation and Oil Red O Staining

Fresh tissue was fixed in Paraformaldehyde (4%,w/v) for more than 24 h. The tissue was removed from the fixed solution and dehydrated. OCT embedding agent was dropped around the dehydrated tissue and the tissue was placed on a quick freezing table. As the OCT embedded tissue became white and hard, it was sectioned to 8-10 μm thick slice and stored at -20 °C for future use. The frozen slice was reheated, dried and stained with Oil Red solution in the dark. After differentiation, slice was stained with Hematoxylin solution, differentiation solution, and Scott Tap Bluing solution following a standard method. Finally, slice was sealed with glycerin gelatin before analysis.

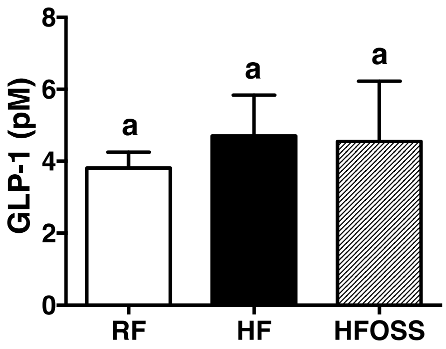

Figure S1. Plasma GLP-1 level of mice fed a regular-fat diet (RF), high-fat diet (HF) and high-fat diet supplemented with OS starch (HFOSS). Values with different lowercase letters indicate significant differences between groups (P<0.05).

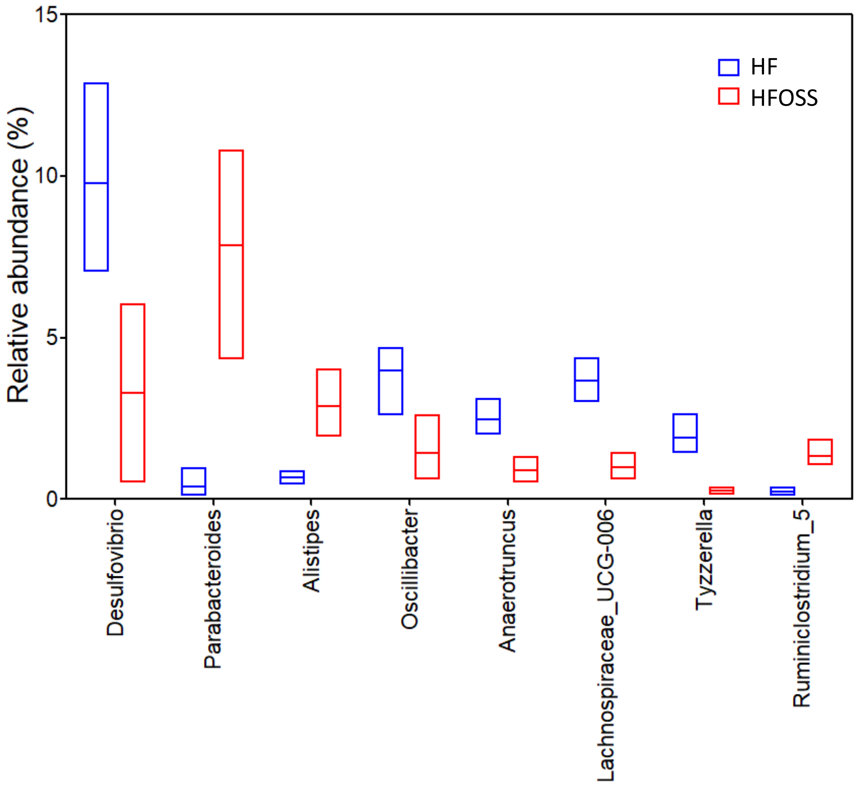

Figure S2. Differential analysis of genus composition between the high-fat diet (HF) group and high-fat diet supplemented with OS starch (HFOSS) group. Each box diagram shows the minimum, first quartile, medium, third quartile and maximum values of the index of the corresponding group.

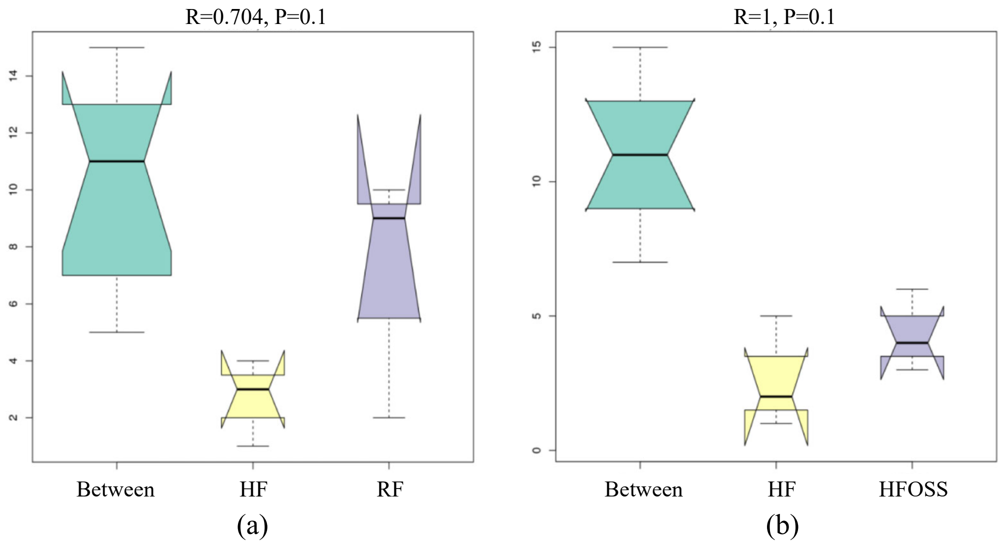

Figure S3. Group comparison by Anosim analysis. (a) Anosim analysis of mouse fecal microbiota between the high-fat diet (HF) group and regular-fat diet (RF) group. (b) Anosim analysis of mouse fecal microbiota between the HF group and the high-fat diet supplemented with OS starch (HFOSS) group.
